# Genetic and wind field analysis of wheat leaf rust (*Puccinia triticina*) dispersal: from winter sources in Gansu and Shaanxi to summer epidemics in China

**DOI:** 10.3389/fpls.2025.1558898

**Published:** 2025-05-14

**Authors:** Hongfu Li, Na Zhao, Qinqin Zhang, Liang Huang, Hao Zhang, Li Gao, Wanquan Chen, Taiguo Liu

**Affiliations:** ^1^ State Key Laboratory for the Biology of Plant Diseases and Insect Pests, Institute of Plant Protection, Chinese Academy of Agricultural Science, Beijing, China; ^2^ National Agricultural Experimental Station for Plant Protection, Ministry of Agriculture and Rural Affairs, Gangu, Gansu, China; ^3^ Control of Biological Hazard Factors (Plant Origin) for Agri-product Quality and Safety, Ministry of Agriculture and Rural Affairs, Beijing, China

**Keywords:** wheat leaf rust, *Puccinia triticina*, population genetic structure, pathogen dispersal, genetic relationship

## Abstract

Wheat leaf rust caused by *Puccinia triticina* (*Pt*) is one of the most serious diseases affecting wheat worldwide. Given that China is the world’s largest wheat-producing country, there is a lack of comprehensive understanding regarding the temporal and spatial dynamics of wheat leaf rust epidemics. This study investigated the population structure of *Pt* across different wheat leaf rust epidemic seasons in Gansu and Shaanxi provinces. Samples were collected in the 2020 winter from Gansu and Shaanxi, and during the 2021 spring and summer from eight additional provinces: Shandong, Henan, Gansu, Shaanxi, Hubei, Yunnan, Guizhou, and Xinjiang. Population genetics analysis was conducted using 12 pairs of Simple sequence repeat (SSR) markers. The results indicated that the genetic diversity of the samples was highest in Shandong (SD) and Shaanxi in the 2020 winter (SN_20w), and Shaanxi in the 2021 summer (SN_21s), while Xinjiang (XJ) exhibited the lowest genetic diversity. Population structure analysis revealed six distinct genetic backgrounds across the 10 populations, with SN_20w and XJ showing greater genetic distances compared to other populations. There was less genetic differentiation and strong gene flow between pairwise populations of Henan (HA) and SN_21s, SD and Gansu in the winter of 2020 (GS_20w), Gansu in the summer of 2021 (GS_21s) and Hubei (HB), and GS_20w and HB, with six shared multi-locus genotypes detected among four pairwise populations. Integrating population genetic analysis, horizontal wind field analysis, topographic analysis, and the sampling timeline, this study concluded that the winter populations of *Pt* in the Guanzhong region experienced dominance shifts, with limited impact on the wheat leaf rust epidemic of 2021. In the 2021 epidemic season, two pathways of pathogen dispersal were proposed: (1) from the Guanzhong basin to Henan province; (2) through the Hanshui River Valley to Hubei province. These findings provide valuable insights into the spatial dynamics of wheat leaf rust and inform targeted prevention and control strategies.

## Introduction

Wheat leaf rust caused by *Puccinia triticina* (*Pt*) is one of the most significant diseases affecting global wheat production, resulting in the highest wheat yield losses worldwide (Prasad et al., 2020; Savary et al., 2019). According to an estimate proposed by an ecoclimatic suitability model, 94.4% of global wheat production is vulnerable to wheat leaf rust, with an estimated annual average loss of 8.6 to 18.3 million tons worldwide between 2000 and 2050 (Chai et al., 2020). China is the world’s largest wheat-producing country, experiencing substantial annual wheat production losses of approximately 3 million tons due to wheat leaf rust (FAOSTAT, 2022; Huerta-Espino et al., 2011). Identifying resistance genes, breeding resistant varieties, and deploying these varieties across wheat-growing regions with diverse climatic conditions represent cost-effective and efficient measures to combat wheat leaf rust.

To address the disease, it is crucial to monitor disease structure and identify key factors affecting disease transmission and prevalence before implementing resistance breeding strategies. Annual race identification of the wheat leaf rust pathogen based on single leaf-rust-resistance genes has provided insights into the pathogen’s virulence structure (Li et al., 2024; Liu and Chen, 2012; Zhang et al., 2020). Additionally, molecular marker-based population structure analysis has revealed genetic relationships among populations across different wheat production areas.

Studies have been conducted to uncover genetic relationships and pathogen dispersal between different *Pt* populations during a single epidemic season. Xu et al. (2022) collected 622 isolates of *Pt* from 15 provinces, municipalities, and autonomous regions in China in 2018. Their research clarified genetic relationships among pathogen populations in the main wheat-producing areas and suggested that the spread of *Pt* follows an east-to-west and south-to-north direction during the disease epidemic season in China. Focusing on *Pt* populations in the eastern wheat-producing regions of China, Li et al. (2023) identified differences between northern and southern populations, which were bounded by the Qinling Mountains-Huaihe River line.

Under natural conditions, *Pt* completes its life cycle through repeated infections via the asexual reproduction of urediniospores, with the overwintering and oversummering of pathogens playing a crucial role in disease epidemics. Fundamental studies have confirmed that *Pt* can overwinter on winter wheat and volunteer wheat in areas south of Shijiazhuang city, Hebei province (Bu et al., 1953; Bu and Zhou, 1952; Zeng et al., 1957). A zonation study based on the termination temperature threshold for urediniospores of *Pt* revealed an oversummering zone spanning from the southwest to the northeast, encompassing Yunnan, western Guizhou, central Sichuan, southeast Gansu, central and northern Shaanxi, southern Ningxia, most of Shanxi, central Inner Mongolia, and western and northern Hebei (Xia, 2021).

In practical studies of wheat stripe rust, regions such as Longnan and Tianshui in Gansu and Baoji in Shaanxi are recognized as important autumn sources of inoculum. The wheat stripe rust pathogen can overwinter in the form of hyphae in these regions and infect autumn-sown wheat, serving as the initial inoculum in the following spring, subsequently spreading to susceptible wheat in the eastern wheat-producing areas (Chen et al., 2013; Zhao and Kang, 2023). Geographically, Longnan, Tianshui, and Baoji act as bridges connecting the east and west and the north and south. However, between different wheat leaf rust epidemic seasons, it remains unclear whether pathogen populations in these regions are important sources for the disease prevalence in the following year or how winter sources contribute to the population structure in various wheat-growing regions.

To clarify the genetic relationship between winter populations of *Pt* in Gansu and Shaanxi provinces and the epidemic populations in the main wheat areas of China in the following year, samples were collected from Longnan and Tianshui in Gansu province and Baoji in Shaanxi province in the winter of 2020, and samples from Shandong, Henan, Hubei, Gansu, Shaanxi, Guizhou, Yunnan and Xinjiang in China were collected in the epidemic season of the following year (2021) to conduct population genetic structure analysis. This study aims to enhance our understanding of the annual dynamics of wheat leaf rust disease within the existing knowledge of population genetic structure and is vital for gaining a comprehensive insight into wheat leaf rust epidemic patterns.

## Materials and methods

### *Puccinia triticina* sampling

**Figure 1:**
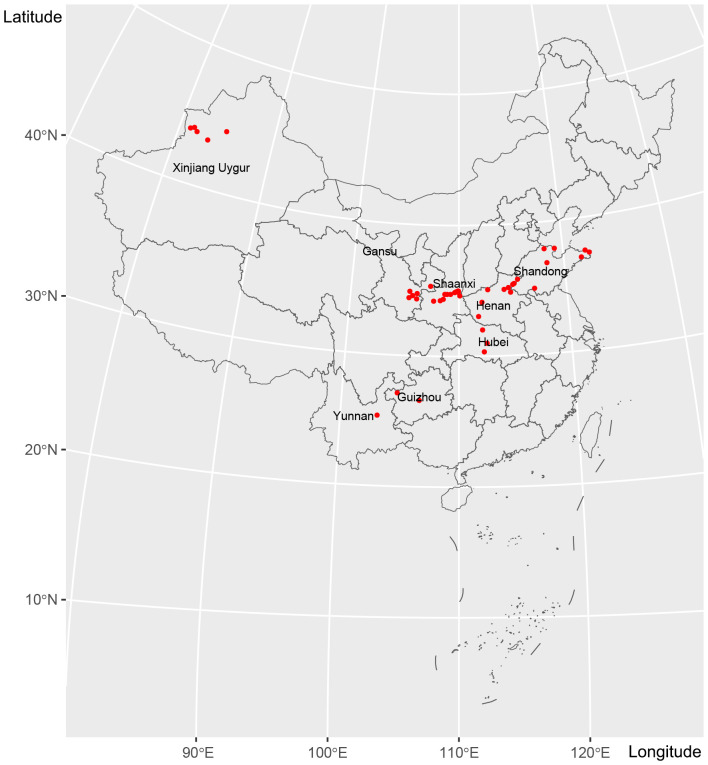
A total of 199 *Pt* samples were collected from 49 sampling sites in eight provinces and autonomous regions of China from winter 2020 to summer 2021.

In this study, a total of 199 *Pt* samples were collected across 49 sampling sites (**Figure 1**; **Table 1**). In November and December 2020, 40 *Pt* samples were collected from Gansu and Shaanxi provinces. Subsequently, from March to June 2021, 159 *Pt* samples were collected from Shandong, Henan, Gansu, Shaanxi, Hubei, Yunnan, Guizhou, and Xinjiang. Wheat leaf samples were wrapped in craft paper and stored at 4°C. Sampling sites were visualized using *sf* v 1.0-18, *tidyverse* v 2.0.0, *maptools* v 1.1-5, and *rmapshaper* v 0.5.0 in R (Bivand and Lewin-Koh, 2022; Pebesma, 2018; Teucher and Russell, 2023; Wickham H et al., 2019).

**Table 1:** Sampling information of *Pt* from 10 populations in eight provinces and autonomous regions of China from 2020 to 2021.

Province	Population	City	Sampling date	Isolates number	Sampling altitude (m)	Population size
Shandong	SD	Weihai	2021.3.2–2021.4.27	8	49–90	16
		Zibo	2021.4.23	2	41	
		Binzhou	2021.4.26	4	3–4	
		Zaozhuang	2021.4.26	2	47–92	
Henan	HA	Luoyang	2021.4.12	7	296	25
		Nanyang	2021.4.10	4	200	
		Puyang	2021.5.17–2021.5.19	6	61–106	
		Xinxiang	2021.5.17–2021.5.19	4	72-77	
		Jiyuan	2021.5.17–2021.5.19	2	133	
		Kaifeng	2021.5.17–2021.5.19	2	75	
Shaanxi	SN_20w	Baoji	2020.12.24	21	630	21
	SN_21s	Weinan	2021.5.31	24	382–574	34
		Xianyang	2021.5.21	10	525–608	
Gansu	GS_20w	Longnan	2020.11.19	7	1,674	19
		Tianshui	2020.11.17–2020.11.18	12	1,566–1,749	
	GS_21s	Tianshui	2021.6.12	2	1,377	7
		Pingliang	2021.6.11	5	1,042	
Hubei	HB	Jingzhou	2021.4.13	6	/	13
		Xiangyang	2021.5.28	7	60	
Yunnan	YN	Kunming	2021.3.5	21	1,914	21
Guizhou	GZ	Bijie	2021.6.16	5	2,209	17
		Guiyang	2021.5.20	12	/	
Xinjiang	XJ	Yili	2021.6.25	26	602–966	26
Total						199

### SSR amplification

For SSR amplification, a 1 cm^2^ leaf segment with a single uredinium of *Pt* was placed in a 2 mL grinding tube, along with one grain of steel beads (YA3032–500 g, Beijing Solarbio Technology Co., Ltd., China) and 0.3 g of 1.0 mm glass grinding beads (BE6061-500, Beijing Easybio Technology Co., Ltd., China). These were frozen in liquid nitrogen and then ground at 1,800 cycles/min for 30 s using a Fastprep tissue homogenizer (MpBio, China). DNA was extracted using a plant genomic DNA kit (TIANGEN Biotech Co. Ltd., Beijing, China). The DNA concentration and purity were assessed using a DS-11FX ultra-micro/fluorescence full-function spectrophotometer (DeNovx), with samples with a DNA concentration >20 ng/μL selected for SSR amplification.

In this study, 12 pairs of fluorescent SSR primers, including RB8, RB1, RB35, RB11, and RB16 (Duan et al., 2003), and PtSSR151A, PtSSR161, PtSSR91, PtSSR152, PtSSR92, PtSSR173, and PtSSR13 (Szabo and Kolmer, 2007) (**Supplementary Table S1**) (Beijing Tsingke Biotech Co., Ltd., China), were utilized for SSR amplification. Each PCR reaction was set up with a 12.5 μL mixture containing 6.25 μL of Taq DNA mix (2X M5 Hiper plus Taq HiFi PCR mix, Beijing Mei5 Biotechnology Co., Ltd., China), 0.5 μL of each primer, 0.5 μL of DNA template, and 4.75 μL of deionized water. The PCR procedure involved an initial denaturation at 95°C for 3 min, followed by 35 cycles of 94°C for 25 s, annealing at 58°C–61°C for 15 s, and elongation at 72°C for 5 min, with a final extension at 72°C for 10 min.

PCR products were diluted at 1:50 with deionized water and loaded into a 96-well plate. Each well received 1 μL of the diluted PCR product and 9 μL of Hi-Di Formamide (Applied Biosystems, Thermo Fisher Scientific, USA) and GeneScan 500 Lizi (Applied Biosystems, Thermo Fisher Scientific, USA) mixture (at a ratio of 1000:15). The mixture was heated for 5 min at 95°C, followed by an ice bath for 3 min. The length detection of SSR amplification was performed using a 3500 Genetic Analyzer (Applied Biosystems, Thermo Fisher Scientific, USA), with fragment sizes identified using GeneMarker software v 2.7.0 (SoftGenetics, LLC., USA).

### Population genetic structure

Preliminary data analyses were conducted using the R package *poppr* v 2.9.3 (Kamvar et al., 2015, 2014; Team, 2024). Missing values for each SSR locus were less than 10%, and missing values for each sample were less than 25%. Genetic polymorphisms of SSR loci and gene accumulation curves were calculated using *poppr* v 2.9.3. Clone correction was performed to eliminate potential bias caused by cloned genotypes.

Genetic background analysis was conducted using STRUCTURE without prior assignment of samples into distinct populations (Evanno et al., 2005). Parameters were set with a burn-in period of 10,000, followed by 100,000 MCMC repetitions, and K values ranging from 2 to 10 with 10 independent runs. The results were processed using StructureSelector to identify the most appropriate K value (Li and Liu, 2018), and a graphical representation for the specific K was generated using StructureSelector, classifying samples into different populations.

Discriminant analysis of principal components (DAPC) and clustering analyses were performed on data post-clone correction. DAPC was conducted using *adegenet* v 2.1.10 in R (Jombart, 2008), with visualizations performed using *ade4* v 1.7-22 (Dray and Dufour, 2007). Clustering analysis was performed using *ape* v 5.8 in R with Nei’s genetic distance (Paradis and Schliep, 2019), and visualizations were generated using *ggplot2* v 3.5.1 (Wickham, 2016).

Arlequin v.3.5.2.2 was used to calculate the F-statistics (*F_ST_*) with the number of permutations = 10000 (Excoffier et al., 2005). GENALEX 6.5 software was employed to calculate gene flow (*Nm*) between different populations (Peakall and Smouse, 2012; Slatkin, 1985; Wright, 1951). Shared multi-locus genotypes (MLGs) between populations were analyzed using *poppr* v 2.9.3 in R.

### Wind driving force on pathogen dispersal

Horizontal wind field analysis was performed using *xarray* v 2022.3.0 (Hoyer and Hamman, 2017), *pandas* v 2.0.0 (https://pandas.pydata.org/), *numpy* v 1.22.0 (Harris et al., 2020), *cartopy* v 0.23.0 (https://scitools.org.uk/cartopy/docs/latest/), and *matplotlib* v 3.8.0 (Hunter, 2007) in Python v 3.9.7. ERA5 (ECMWF Reanalysis v5) hourly data on pressure levels from 1940 to present were utilized to extract the u-component and v-component of wind in January and February 2021 (Hersbach et al., 2023). Considering the altitude range of the sampling sites in Shaanxi (382–630m) and Gansu (1,042–1,749m), and the time period between winter and summer sampling, ERA5 hourly data on pressure levels of 950hPa and 850hPa were extracted for horizontal wind field analysis in January and February 2021.

MeteoInfoMap v 3.9.6 (Wang, 2014) was used to analyze the airflow trajectories at several sampling sites to supplement the horizontal wind field analysis. The meteorological data were downloaded from the NOAA (https://www.noaa.gov/). Specifically, the forward airflow trajectories of Longnan (2020.11.25–2020.11.27) in Gansu province, backward airflow trajectories of Jingzhou (2021.3.22–2021.3.24) in Hubei province, backward airflow trajectories of Xiangyang (2021.5.6–2021.5.9) in Hubei province, backward airflow trajectories of Luoyang (2021.3.27–2021.3.28) in Henan province, and backward airflow trajectories of Jiyuan (2021.5.6–2021.5.8) in Henan province were examined. The airflow trajectories were generated hourly and analyzed over 48 h. Cluster calculations were applied to group the trajectories, and the airflows in different directions were visually distinguished by assigning different colors based on the proportion of each airflow trajectory.

### Topographic analysis

Topographic analysis was conducted on the sampling sites of six populations in Gansu, Shaanxi, Henan, and Hubei provinces to explore topographic barriers and potential pathways of pathogen dispersal. MeteoInfoMap v 3.9.6 software was used to label sampling sites by latitude and longitude on satellite maps, with BingSatelliteMap serving as the base map (Wang, 2014).

## Results

### Sampling sites and populations

The 199 isolates collected from 2020 to 2021 were divided into 10 populations based on seasonal differences and sampling locations (**Figure 1**; **Table 1**). SN_20w (winter 2020, Baoji, Shaanxi) and GS_20w (winter 2020, Longnan and Tianshui, Gansu) represented winter samples. The remaining eight populations, all sampled in 2021, were classified according to their geographic locations: SD (Shandong), HA (Henan), SN_21s (Shaanxi, summer 2021), GS_21s (Gansu, summer 2021), HB (Hubei), YN (Yunnan), GZ (Guizhou), and XJ (Xinjiang).

### Genetic polymorphism

The genetic polymorphism of the 12 pairs of SSR markers used in this study revealed a range of allele numbers from 2 to 9, with an average of 5.58 (**Table 2**). The Simpson's index (*1-D*) and Nei’s gene diversity (*He*) both averaged 0.53, indicating that the selected markers provided appropriate polymorphism for this sample set. The evenness values ranged from 0.44 to 0.88, suggesting a reasonably balanced distribution of allele frequencies within the sample set.

**Table 2:** Polymorphism indexes of 12 pairs of SSR markers used in this study.

Locus	Allele	*1-D*	*He*	Evenness
RB8	5	0.61	0.61	0.75
RB1	3	0.51	0.51	0.79
RB35	8	0.65	0.65	0.64
PtSSR151A	6	0.55	0.55	0.68
PtSSR161	8	0.72	0.72	0.71
RB11	4	0.62	0.62	0.88
PtSSR91	2	0.07	0.07	0.45
PtSSR152	5	0.53	0.53	0.82
PtSSR92	8	0.54	0.54	0.50
PtSSR173	9	0.77	0.78	0.80
PtSSR13	5	0.61	0.61	0.70
RB16	4	0.12	0.12	0.44
Mean	5.58	0.53	0.53	0.68

*1-D*, Simpson's Index; *He*, Nei’s gene diversity.

The 199 isolates generated a total of 170 MLGs using the 12 pairs of SSR primers, and the gene accumulation curve indicated that this set of primers provided adequate discrimination for the sample set (**Supplementary Figure S1**). Among the 10 populations divided by sampling seasons and sampling sites, only GS_21s exhibited a population size of less than 10. The populations SD, SN_20w, and SN_21s exhibited the highest level of eMLG (10.00), suggesting a greater diversity of genotypes within these populations. In contrast, the XJ population had the lowest *uHe* value (0.37), indicating the lowest genetic diversity among the populations studied (**Table 3**).

**Table 3:** Population polymorphism indexes of 10 *Pt* populations.

No.	Pop	*N*	MLG	eMLG	*H*	*1-D*	*uHe*
1	SD	16	16	10.00	2.77	0.94	0.47
2	HA	25	21	9.30	2.98	0.94	0.43
3	SN_20w	21	21	10.00	3.04	0.95	0.52
4	SN_21s	34	34	10.00	3.53	0.97	0.44
5	GS_20w	19	15	8.81	2.63	0.92	0.47
6	GS_21s	7	7	7.00	1.95	0.86	0.44
7	HB	13	12	9.42	2.46	0.91	0.46
8	YN	21	16	8.80	2.69	0.93	0.43
9	GZ	17	14	9.01	2.59	0.92	0.51
10	XJ	26	20	8.92	2.88	0.94	0.37
11	Total/Mean	199	170	9.91	5.06	0.99	0.53

*N*, number of samples; MLG, multi-locus genotype; eMLG, number of expected MLGs at the smallest sample size ≥ 10 based on rarefaction; *H*, Shannon–Wiener index of MLG diversity; *1-D*, Simpson’s index; *uHe*, Nei’s unbiased gene diversity.

### Population genetic structure

**Figure 2:**
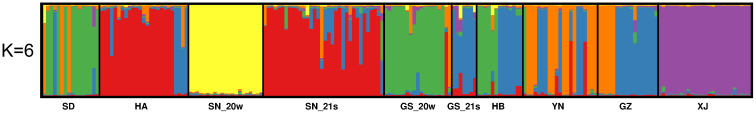
The genetic structure analysis of 10 *Pt* populations based on the optimal K value (K=6) revealed the relationships between genetic backgrounds among the populations.

A total of 199 samples without pre-defined populations using STRUCTURE analysis yielded an optimal K value of 6 (**Figure 2**; **Supplementary Table S1**). The genetic backgrounds of the SN_20w and XJ populations were distinct from other populations. The HA population showed a similar genetic background to SN_21s, and the two populations were geographically adjacent at sampling sites. The genetic lineages of HB were primarily composed of the main genetic lineages of GS_20w and GS_21s, suggesting a frequent dispersal of strains between the Hubei and Gansu populations in different seasons. The YN and GZ populations exhibited genetic lineages distinct from other regions (represented by the orange bar block), while the wheat leaf rust pathogens in these two regions had close genetic relationships. The SD population exhibited a mixed genetic background, with its main genetic lineage (represented by the green bar block) found in the GS_20w and HB populations, and other genetic lineages (represented by the blue and orange bar blocks) also present, indicating that the SD population may have been an introduced population with multiple sources of pathogen origins.

**Figure 3:**
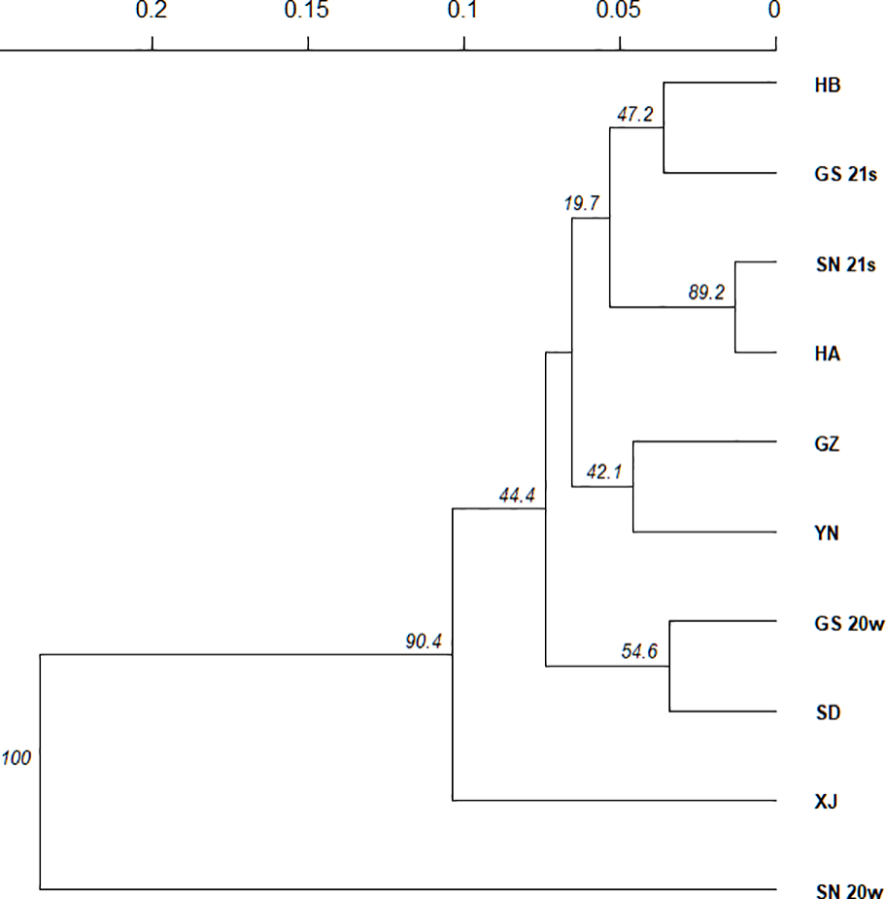
Phylogenetic analysis based on Nei’s genetic distance for 10 *Pt* populations.

The phylogenetic analysis based on Nei’s genetic distance for the 10 populations supported the results of the STRUCTURE analysis (**Figure 3**). SN_20w was completely separated from the other populations, indicating genetic distinctiveness and possibly a different origin. The XJ population was also genetically independent. Among the remaining eight populations, the pairwise populations of SD and GS_20w, GZ and YN, SN_21s and HA, and HB and GS_21s each showed closer genetic relationships with each other than the others.

**Figure 4:**
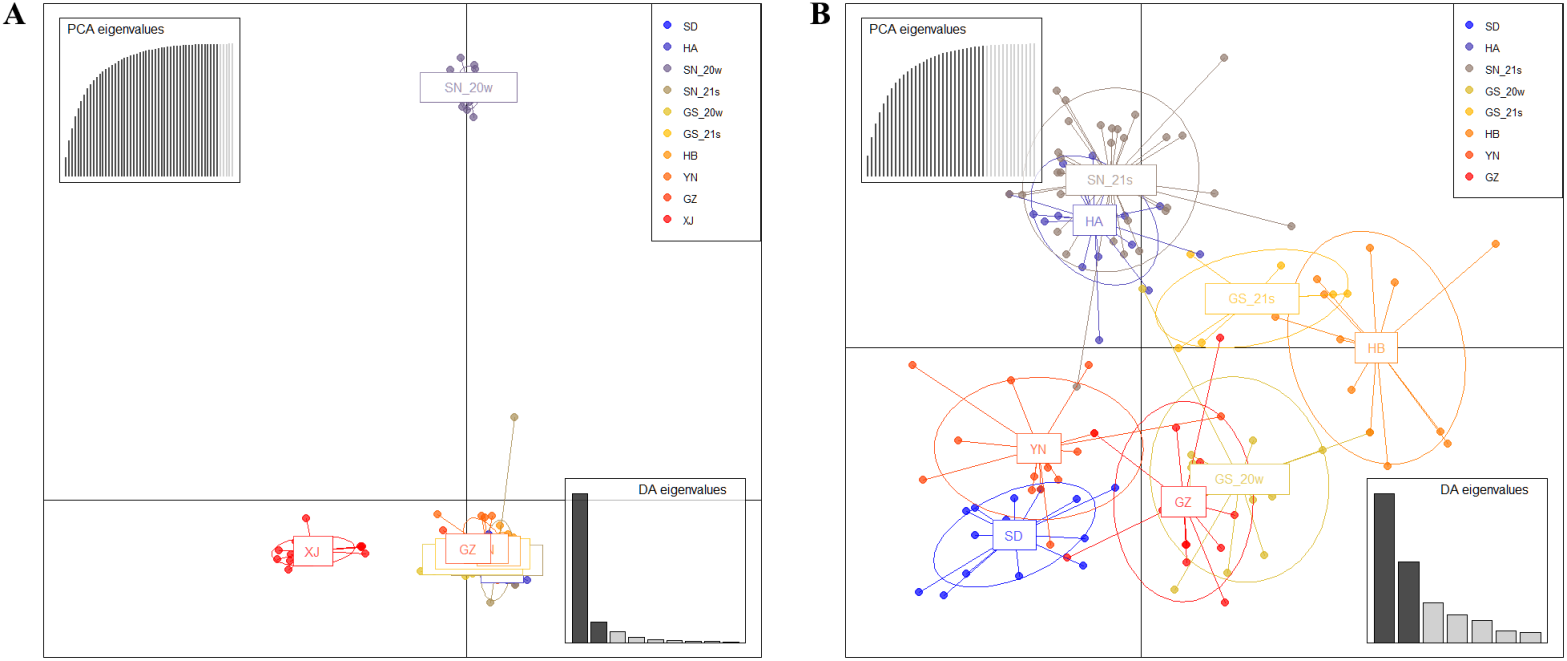
Discriminant analysis of principal components of the 10 *Pt* populations **(A)** and eight populations excluding XJ and SN_20w **(B)**.

In the DAPC of the 10 populations, the first eigenvalue obtained from the DA analysis was much higher than the rest, indicating that this eigenvalue explained a large part of the variation between populations, demonstrating significant genetic differentiation of SN_20w and XJ populations from the other populations (**Figure 4A**). In the DAPC for the remaining eight populations, since the SD population was geographically distant from the YN and GZ populations, the close genetic relationship might be related to the long-distance spread of *Pt* (**Figure 4B**). The HB population was genetically distinct from the other populations but closely related to GS_20w and GS_21s, indicating potential genetic exchange between the Hubei and Gansu populations despite their geographic separation.

### Genetic differentiation and gene flow

The fixation index (*F_ST_*) and gene flow (*Nm*, Nei’s migration) parameters between pairwise populations were calculated, with results sorted from the highest to the lowest *Nm* values (**Table 4**). The *F_ST_* values reflect varying levels of genetic differentiation: 0 < *F_ST_* < 0.05 indicates low differentiation, 0.05 ≤ *F_ST_* < 0.15 indicates moderate differentiation, *F_ST_* ≥ 0.15 indicates high differentiation, and *F_ST_* ≥ 0.25 indicates higher differentiation among populations. Notably, pairwise populations with *F_ST_* values less than 0.05 (HA and SN_21s, SD and GS_20w, GS_21s and HB, GS_20w and HB) exhibited strong gene flow (*Nm* > 4), suggesting reduced genetic differentiation due to increased genetic exchange between these populations. Focusing on the winter and summer epidemic populations in the Gansu and Shaanxi regions, the summer populations (SN_21s and GS_21s) showed moderate genetic differentiation (*F_ST_* = 0.051), while the winter populations (SN_20w and GS_20w) exhibited high genetic differentiation (*F_ST_* = 0.208). For populations within the same region but different seasons, GS_20w and GS_21s showed moderate genetic differentiation (*F_ST_* = 0.057), and SN_20w and SN_21s showed high differentiation (*F_ST_* = 0.211). These patterns suggested that the summer epidemic populations in the two regions might not have been directly caused by continuous infection from the winter populations.

**Table 4:** The *F_ST_* (fixation index) and gene flow (*Nm*, Nei’s migration) parameters between pairwise *Pt* populations.

Pop1	Pop2	*F_ST_*	*P*	*Nm*	Pop1	Pop2
HA	SN_21s	0.015	0.009 ± 0.001	14.872	25	34
SD	GS_20w	0.047	0.001 ± 0.000	5.196	16	19
GS_21s	HB	0.010	0.025 ± 0.002	4.826	7	13
GS_20w	HB	0.035	0.001 ± 0.000	4.579	19	13
SN_21s	GS_21s	0.051	0.003 ± 0.001	4.481	34	7
HA	GS_21s	0.071	0.001 ± 0.000	3.268	25	7
YN	GZ	0.067	0.002 ± 0.001	2.97	21	17
GS_21s	GZ	0.078	0.005 ± 0.001	2.857	7	17
SD	YN	0.053	0.004 ± 0.001	2.501	16	21
SN_21s	YN	0.107	0.000 ± 0.000	2.009	34	21
HA	YN	0.108	0.000 ± 0.000	1.969	25	21
SN_21s	HB	0.093	0.000 ± 0.000	1.967	34	13
SN_21s	GZ	0.115	0.000 ± 0.000	1.957	34	17
GS_20w	GZ	0.079	0.000 ± 0.000	1.937	19	17
HA	GZ	0.121	0.000 ± 0.000	1.923	25	17
SD	GZ	0.093	0.000 ± 0.000	1.92	16	17
HB	GZ	0.120	0.000 ± 0.000	1.837	13	17
GS_20w	YN	0.070	0.000 ± 0.000	1.799	19	21
GS_20w	GS_21s	0.057	0.002 ± 0.000	1.791	19	7
SD	HB	0.130	0.000 ± 0.000	1.734	16	13
HA	HB	0.116	0.000 ± 0.000	1.695	25	13
HA	GS_20w	0.070	0.000 ± 0.000	1.688	25	19
SN_21s	GS_20w	0.066	0.000 ± 0.000	1.685	34	19
GS_21s	YN	0.139	0.001 ± 0.000	1.672	7	21
HB	YN	0.127	0.000 ± 0.000	1.531	13	21
GZ	XJ	0.130	0.000 ± 0.000	1.462	17	26
SD	SN_21s	0.104	0.000 ± 0.000	1.443	16	34
SD	HA	0.119	0.000 ± 0.000	1.354	16	25
GS_21s	XJ	0.146	0.000 ± 0.000	1.327	7	26
SD	GS_21s	0.136	0.000 ± 0.000	1.265	16	7
SN_21s	XJ	0.188	0.000 ± 0.000	1.086	34	26
HA	XJ	0.200	0.000 ± 0.000	0.989	25	26
YN	XJ	0.229	0.000 ± 0.000	0.918	21	26
SN_20w	GZ	0.185	0.000 ± 0.000	0.829	21	17
HB	XJ	0.210	0.000 ± 0.000	0.806	13	26
SN_20w	GS_21s	0.184	0.000 ± 0.000	0.779	21	7
GS_20w	XJ	0.194	0.000 ± 0.000	0.762	19	26
SN_20w	GS_20w	0.208	0.000 ± 0.000	0.7	21	19
SN_20w	YN	0.231	0.000 ± 0.000	0.68	21	21
SN_20w	HB	0.230	0.000 ± 0.000	0.674	21	13
SD	XJ	0.244	0.000 ± 0.000	0.664	16	26
HA	SN_20w	0.212	0.000 ± 0.000	0.66	25	21
SN_20w	SN_21s	0.211	0.000 ± 0.000	0.65	21	34
SD	SN_20w	0.255	0.000 ± 0.000	0.637	16	21
SN_20w	XJ	0.294	0.000 ± 0.000	0.483	21	26

The results were sorted from the highest to the lowest according to the *Nm* values, and the *P*-values indicated that there were significant differences between pairwise *F_ST_* at *P*<0.05.

### Shared MLGs

Shared MLGs provided evidence for pathogen dispersal. Among the 170 MLGs analyzed, six were detected in different populations (**Table 5**). Three shared MLGs were detected between SN_21s and HA populations, while one shared MLG was detected between GS_20w and HB, YN and GZ, and SD and YN populations, respectively. The detection of shared MLGs between the SD and YN populations, which are geographically distant, supported the possibility of long-distance pathogen dispersal events between these two regions. In this study, shared MLGs were only found between pairwise populations, and no MLG was detected in multiple populations. This suggested that inter-regional transmission of *Pt* might also be constrained by factors such as topographic barriers, host adaptability of strains, or different climatic conditions.

**Table 5:** Six shared multi-locus genotypes (MLGs) in pairwise *Pt* populations were detected from 170 MLGs.

MLG	SD	HA	SN_20w	SN_21s	GS_20w	GS_21s	HB	YN	GZ	XJ	Total
MLG 51	1	0	0	0	0	0	0	1	0	0	2
MLG 62	0	0	0	0	3	0	1	0	0	0	4
MLG 89	0	3	0	1	0	0	0	0	0	0	4
MLG 109	0	0	0	0	0	0	0	2	2	0	4
MLG 141	0	1	0	1	0	0	0	0	0	0	2
MLG 143	0	1	0	1	0	0	0	0	0	0	2

### Wind driving force on pathogen dispersal

**Figure 5:**
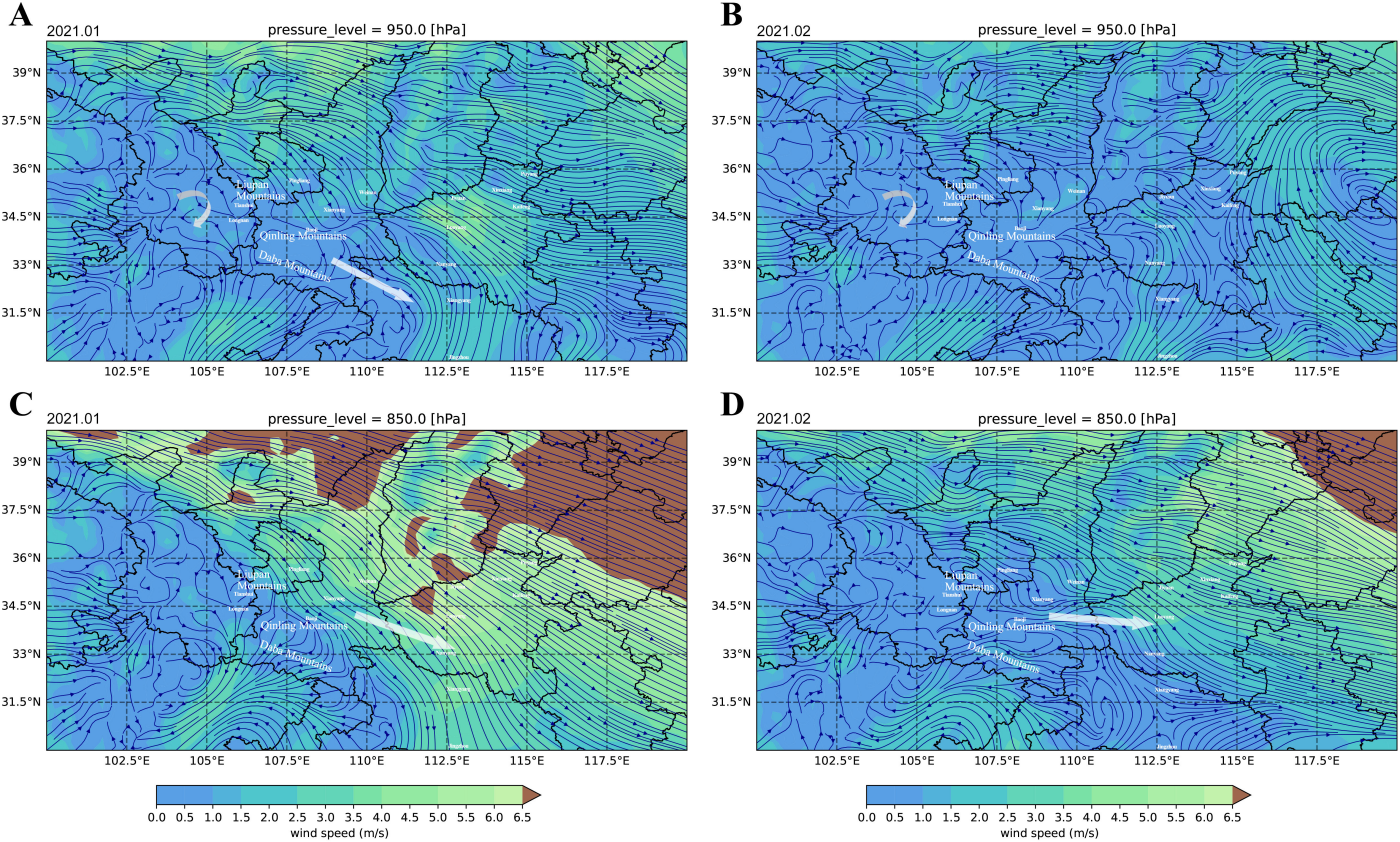
The horizontal wind field analysis showed the potential driving force of wind on urediniospore dispersal. The average wind field of 950 hPa on January 2021 **(A)** and February 2021 **(B)** revealed that the wind streamline disturbance blocked by mountains in southern Gansu provided favorable conditions for the mixing of different genetic populations of urediniospores. The average wind field of 850 hPa on January 2021 **(C)** and February 2021 **(D)** showed that the wind streamline flowing out of Shaanxi via the Qinling Mountains reached Henan.

Wind plays a critical role in the regional migration of urediniospores of *Pt*. In the southern regions of Gansu province, wind streamlines were disrupted by mountain barriers, leading to localized chaotic disturbances that facilitated the mixing of urediniospores (**Figures 5A, B**). On the north and south sides of the Qinling Mountains and the Daba Mountains, wind streamlines were redirected, with wind along the Qinling Mountains flowing towards Henan, and wind between the Qinling Mountains and Daba Mountains tending to flow towards Hubei (**Figures 5C, D**). At both 950 hPa and 850 hPa, the wind streamline between Gansu and Shaanxi was obstructed by the Liupan Mountains and Qingling Mountains.

The forward airflow trajectories revealed airflow from Longnan, Gansu province, to Hubei province in the winter (**Supplementary Figure S2A**). The backward airflow trajectories revealed that Jingzhou (**Supplementary Figure S2B**) and Xiangyang (**Supplementary Figure S2C**) in Hubei province received airflow from Gansu province, and Luoyang (**Supplementary Figure S2D**) and Jiyuan (**Supplementary Figure S2E**) in Henan province received airflow from Shaanxi province in the summer.

## Discussion

During the spring to summer of 2021 wheat leaf rust epidemic season, genetic relationships among populations could potentially reflect pathogen dispersal events. The YN population was sampled earlier (2021.3.5) compared to the SD population (2021.3.20–2021.4.27). Coupled with evidence from population structure and shared MLGs, this suggested pathogen dispersal from Yunnan to Shandong. Genetic diversity of populations in the efferent population is generally higher than that of the afferent population (Brussard, 1984). However, the YN population displayed lower genetic diversity than the SD population, which can be explained in three ways. (1) A previous study in the eastern wheat-producing area positioned Shandong north of the boundary line for *Pt*’s northward transmission (Li et al., 2023). In this study, genetic structure analysis suggested that the SD population might be an afferent population with multiple sources. Under K=6, SD samples encompassed three distinct genetic backgrounds, explaining the higher genetic diversity in the afferent SD population compared to the efferent YN population. (2) Urediniospores of *Pt* can be dispersed over long distances by airflows, allowing different geographical populations to both receive and transmit pathogens, rather than spreading via a single-point diffusion model like soil-borne diseases. “Genetic diversity” is a common result of host selection, pathogen immigration and emigration, inheritance, variation accumulation, and pathogen population evolution, rather than a determinant of pathogen dispersal. More critical factors may include the host adaptability, disease onset timing, urediniospore quantity, and wind strength and direction. (3) The low genetic diversity in the YN population could be related to the reduced wheat cultivation area in the southwestern region. The reduction affected the effective size of the pathogen population and the accumulation of variations within the population. Additionally, the earlier wheat maturation time and rust occurrence times in the southwestern region, combined with fewer external pathogen introductions due to reduced host availability, contribute to the YN population’s lower genetic diversity.

The Xinjiang Uyghur Autonomous Region is geographically remote from other wheat-producing areas, and the XJ population is also genetically distinct, which is supported by similar studies (Zhang et al., 2024). There was significant genetic differentiation between the XJ and other populations (*F_ST_* = 0.146-0.341), with moderate gene flow (*Nm* > 1). The potential eastward dispersal of pathogens from Xinjiang is likely limited and slow. Thus, the influence of the XJ population on the occurrence and prevalence of wheat leaf rust in the main wheat-growing areas and on the genetic structure is insignificant, and vice versa.

Genetic relationships between the populations across different epidemic seasons may reflect both pathogen dispersal events and the dominant shifts in response to environmental changes. The HB population exhibited a mixed genetic background, sharing backgrounds with both GS_20w and GS_21s. Low genetic differentiation and high gene flow were observed between GS_20w and HB, as well as between GS_21s and HB, while GS_20w and GS_21s did not show a close genetic relationship. This suggested that both winter populations and summer populations from Gansu contributed to the occurrence of wheat leaf rust in Hubei during spring to summer in 2021. However, under conditions of chaotic wind streamlines facilitating urediniospore mixing, the high genetic difference between GS_20w and GS_21s populations suggested that genetic population dominance shifts might occur from winter to summer in Gansu, along with multiple strain introductions from the Gansu population to Hubei. Although Hubei and Gansu are not directly adjacent, the SN population in Shaanxi, directly adjacent to Hubei, showed a higher level of genetic differentiation with the HB population. Integrating wind field analysis, we speculate that areas in Gansu such as Longnan and Tianshui, located west of the Liupan Mountains and between the Daba Mountains and the Qinling Mountains, provide a Hanshui River Valley pathway for pathogen dispersal from Gansu to Hubei.

The HA population was more closely related genetically to the SN_21s population. Geographically, the sampling sites for the SN_21s population, Weinan and Xianyang, located north of the Qinling Mountains, which block pathogen dispersal southward. To the west, the Liupan Mountains also form a topographical barrier. Weinan and Xianyang are situated in the Guanzhong Plain, bordered by the Northern Shaanxi Plateau to the north, with the only eastward route being the Guanzhong Basin leading to Henan. Horizontal wind field analysis indeed revealed wind flowing from Shaanxi via the Qinling Mountains to Henan. Genetic analyses indicated that the SN_20w had minimal impact on disease prevalence in the main wheat-growing areas. In contrast, the SN_21s population could pass through the Guanzhong Plain pathway and enter Henan province, the most extensive wheat cultivation area in China. The abundance of hosts in this region further facilitated pathogen infection and widespread dispersal. It also revealed dominance shifts of populations in Shaanxi, where SN_20w might be replaced by predominant populations in changing environmental conditions caused by seasonal changes, infecting wheat in limited areas without further spread.

The difference between SN_20w and SN_21s may be exaggerated due to the limited sample collection of SN_20w, which failed to fully cover the entire genetic composition of the winter wheat leaf rust population in this region. Nevertheless, the difference between GS_20w and GS_21s showed that even within the same area, pathogen population composition was dynamic, involving the process of disease infection and re-infection, with strain population composition determined by strains occupying dominant ecological niches first. Moreover, the genetic differentiation between the Gansu and Shaanxi populations can be explained by the Liupan Mountains and the Qinling Mountains blocking wind streamlines between the two regions.

In studies on the regional spread of wheat stripe rust, strains spreading from the southwestern region to the lower reaches of the Yangtze River played an important role in the population structure (Huang et al., 2025). However, in the population structure study of wheat leaf rust, the strains in southwest China do not seem to play such a crucial role. Previous studies indicated that during the same epidemic season, gene flow between the Yunnan, Sichuan, and Hubei populations was relatively low (*Nm*, Hubei-Yunnan = 5.029, Hubei-Sichuan = 4.715). In contrast, gene flow between Shaanxi, Gansu, and Hubei showed higher levels (*Nm*, Hubei-Shaanxi = 10.638, Hubei-Gansu = 7.308) (Xu et al., 2022). We believe the main reason is that the wheat leaf rust pathogen adapts to a more moderate temperature (10°C–25°C) than the wheat stripe rust pathogen (Ren et al., 2023). The stripe rust pathogen continually increased in Yunnan during the winter but rarely overwinters in most of the Huang-Huai-Hai Plain, the Guanzhong Plain, and the middle and lower reaches of the Yangtze River (Chen et al., 2013). After the temperature rose, the initial inoculum generated by the early occurrence of stripe rust in Yunnan was able to largely colonize after spreading to Hubei. However, the leaf rust pathogen has a broader range of temperature adaptation. The occurrence of leaf rust in Hubei may be affected by local overwinter sources, and imported sources from Gansu and Shaanxi. Therefore, strains introduced from Yunnan to Hubei do not have such a significant ground-breaking effect, and thus do not play a decisive role in the epidemic of leaf rust in Hubei and the middle and lower reaches of the Yangtze River.

**Figure 6:**
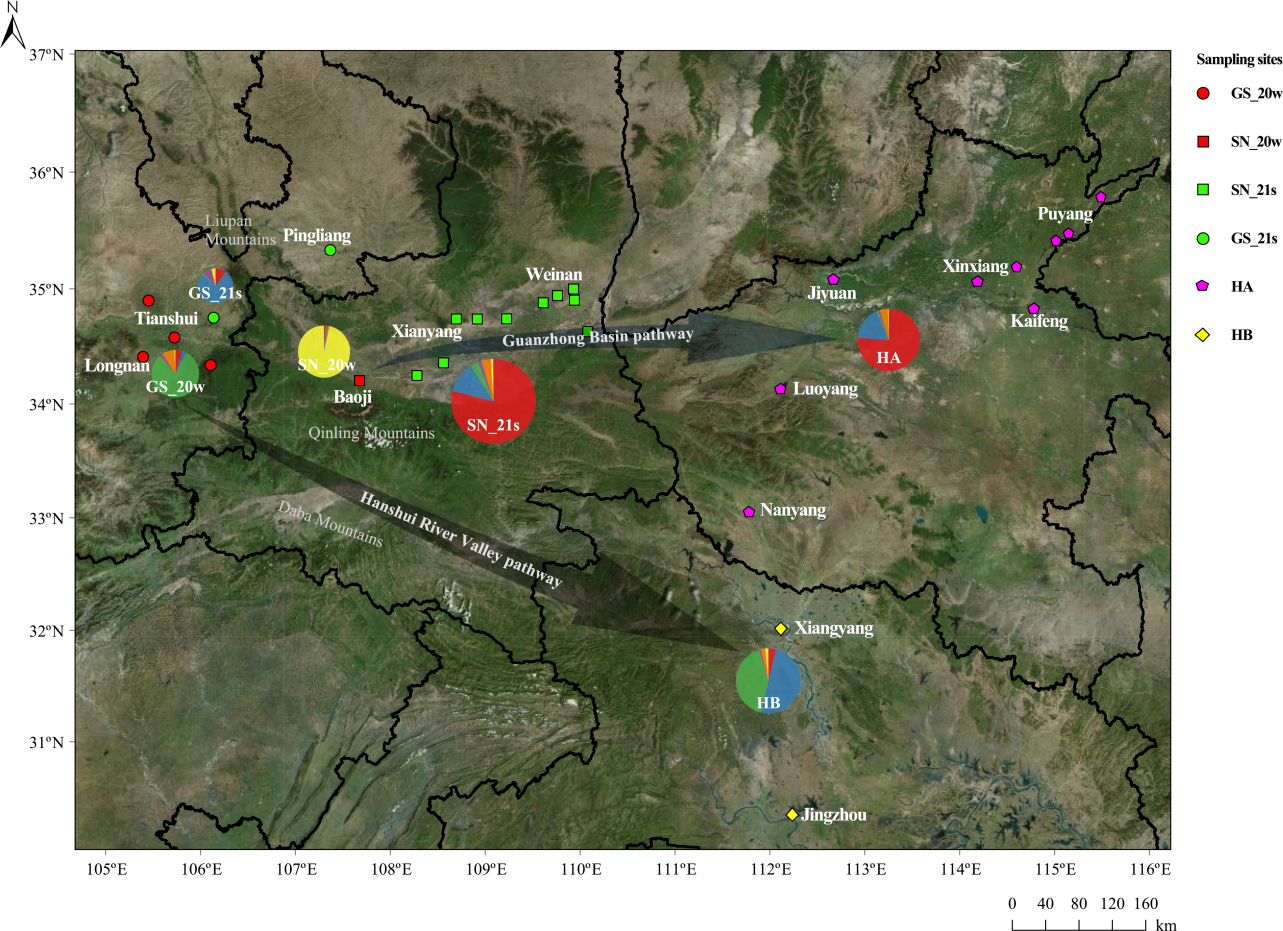
Sampling sites in four provinces of Gansu, Shaanxi, Henan, and Hubei were marked on topographic maps according to different *Pt* populations (different sampling seasons). Arrows indicated two possible pathogen pathways. The pie charts show the population structure of each population, with the same colors as the STRUCTURE analysis.

In summary, the winter populations of *Pt* in the Guanzhong region experienced dominance shifts and had a limited impact on the wheat leaf rust epidemic of 2021. Specifically, GS_20w contributed to the population structure of SD and HB, while SN_20w was not detected during 2021, and the summer populations in Gansu and Shaanxi showed no direct correlation with the winter populations. From winter 2020 to spring 2021, *Pt* from Gansu dispersed to Hubei along the Hanshui River Valley pathway, while the exchange of pathogen sources between Shaanxi and Gansu may have been hindered by the Liupan Mountains and the Qinling Mountains. The winter strains in Shaanxi failed to spread in 2020 and were replaced during dominance shifts. From spring to summer in 2021, strains from Shaanxi were transmitted through the Guanzhong basin pathway to Henan, impacting wheat leaf rust occurrence there. Pathogens from Gansu also followed the Hanshui River Valley pathway into Hubei and potentially spread further to Shandong (**Figure 6**). Shandong province, located north of the boundary line of the northern and southern pathogen populations in the eastern wheat-producing area, received pathogen sources from the western regions of Gansu, Hubei, and Yunnan, and from the southern regions of Jiangsu, Anhui, and Zhejiang. After the pathogens complete multiplication, strains could continue to spread northward, infecting unripe wheat in the North China Plain.

## Data Availability

The original contributions presented in the study are included in the article/**Supplementary Material**, further inquiries can be directed to the corresponding author/s.

## Supplementary material

Supplementary Figure 1 Genotype accumulation curve based on population genetic analysis of 12 SSR markers.

Supplementary Figure 2 Airflow trajectories in several sampling sites, and the ratio of each airflow trajectory showed the proportion of airflow blowing in/from that direction during the time. **(A)** The forward airflow trajectories of Longnan (2020.11.25–2020.11.27) in Gansu province at 1,674m; **(B)** The backward airflow trajectories of Jingzhou (2021.3.22–2021.3.24) in Hubei province at 1,500m; **(C)** The backward airflow trajectories of Xiangyang (2021.5.6–2021.5.9) in Hubei province at 1,500m; **(D)** The backward airflow trajectories of Luoyang (2021.3.27–2021.3.28) in Henan province at 296m; **(E)** The backward airflow trajectories of Jiyuan (2021.5.6–2021.5.8) in Henan province at 133m.

Supplementary Table 1 The K-value parameters of 199 samples obtained using STRUCTURE software.

Supplementary Table 2 The band size of the sample at each SSR locus in the study.
