# Supplementary figures and images for: Genetic and wind field analysis of wheat leaf rust (Puccinia triticina) dispersal: from winter sources in Gansu and Shaanxi to summer epidemics in China
Source: Front Plant Sci. 2025 May 14;16:1558898. doi: 10.3389/fpls.2025.1558898 (PMC12116510; doi:10.3389/fpls.2025.1558898)

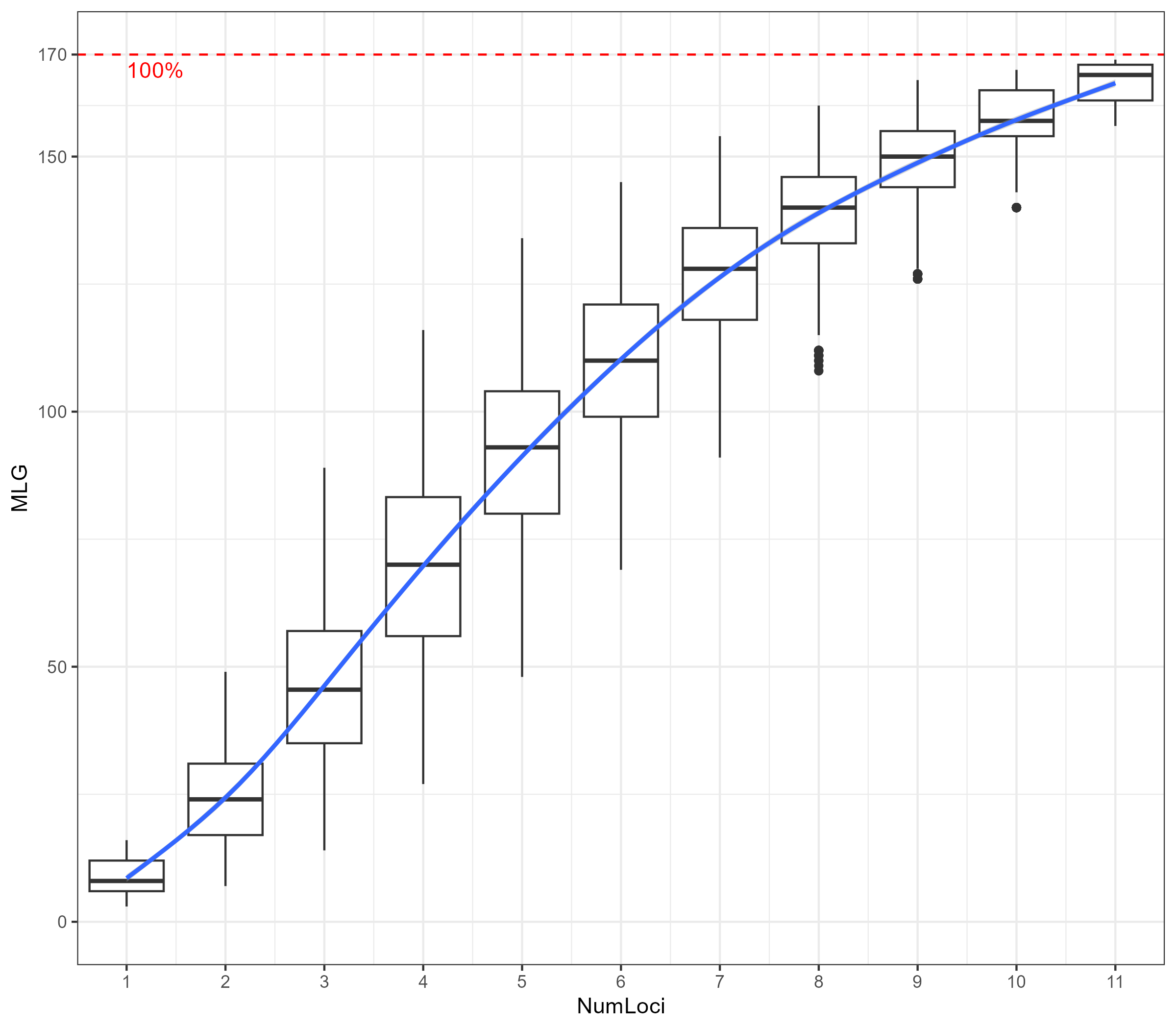

Supplement: Supplementary Figure 1 — Genotype accumulation curve based on population genetic analysis of 12 SSR markers. [file Image1.tiff]

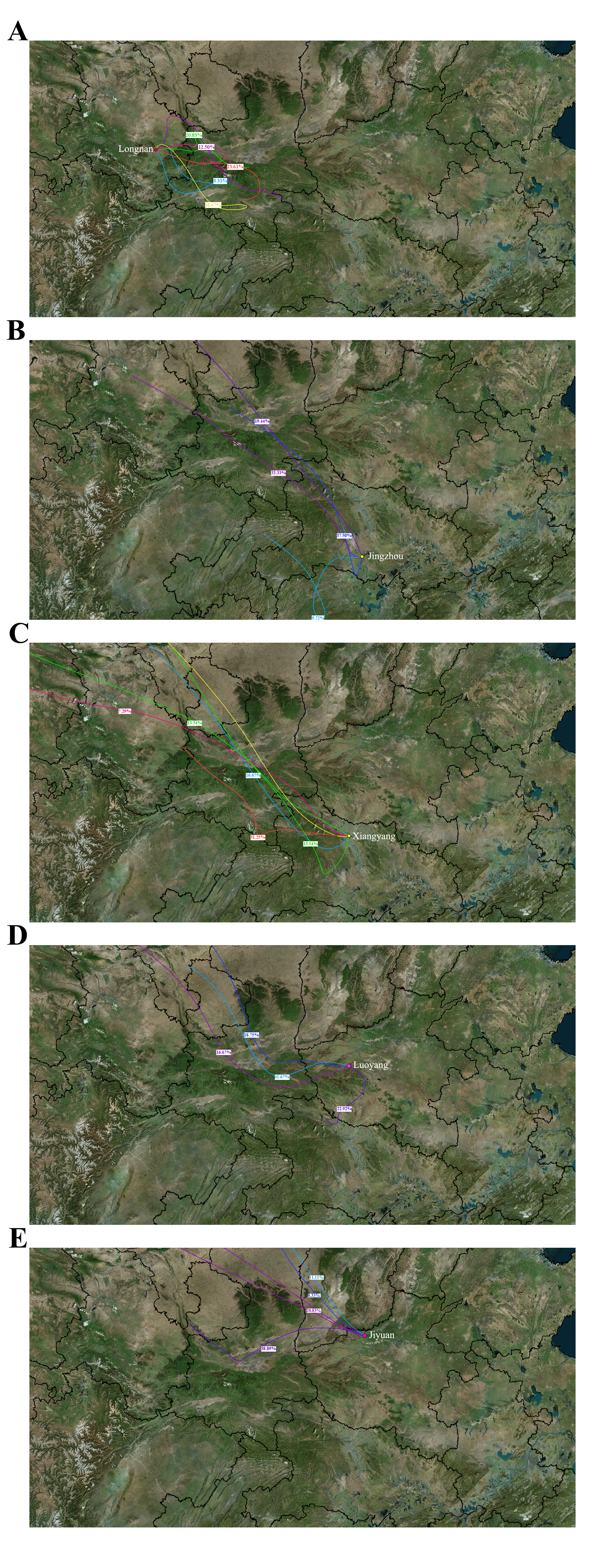

Supplement: Supplementary Figure 2 — Airflow trajectories in several sampling sites, and the ratio of each airflow trajectory showed the proportion of airflow blowing in/from that direction during the time. (A) The forward airflow trajectories of Longnan (2020.11.25–2020.11.27) in Gansu province at 1,674m; (B) The backward airflow trajectories of Jingzhou (2021.3.22–2021.3.24) in Hubei province at 1,500m; (C) The backward airflow trajectories of Xiangyang (2021.5.6–2021.5.9) in Hubei province at 1,500m; (D) The backward airflow trajectories of Luoyang (2021.3.27–2021.3.28) in Henan province at 296m; (E) The backward airflow trajectories of Jiyuan (2021.5.6–2021.5.8) in Henan province at 133m. [file Image2.tif]
